# Potential Markers for Detection and Monitoring of Ovarian Cancer

**DOI:** 10.1155/2011/475983

**Published:** 2011-04-11

**Authors:** Brandon J. D. Rein, Sajal Gupta, Rima Dada, Joelle Safi, Chad Michener, Ashok Agarwal

**Affiliations:** ^1^Center for Reproductive Medicine, Cleveland Clinic, Cleveland, OH 44195, USA; ^2^Laboratory for Molecular Reproduction and Genetics, Department of Anatomy, All India Institute of Medical Sciences, New Delhi 110029, India

## Abstract

This paper reviews current screening techniques as well as novel biomarkers and their potential role in early detection of ovarian cancer. Ovarian cancer is one of the most common reproductive cancers and has the highest mortality rate amongst gynecologic cancers. Because most ovarian cancer diagnoses occur in the late stages of the disease, five-year survival rates fall below 20%. To improve survival rates and to lower mortality rates for ovarian cancer, improved detection at early stages of the disease is needed. Current screening approaches include tumor markers, ultrasound, or a combination. Efforts are underway to discover new biomarkers of ovarian cancer in order to surmount the obstacles in early-stage diagnosis. Among serum protein markers, HE4 and mesothelin can augment CA125 detection providing higher sensitivity and specificity due to the presence of these proteins in early-stage ovarian cancer. Detection testing that includes methylation of the MCJ gene and increased expression of vascular endothelial growth factor is correlated to poor prognosis and may predict patient survival outcome. Detection testing of biomarkers with long-term stability and combination panels of markers, will likely lead to effective screening strategies with high specificity and sensitivity for early detection of ovarian cancer.

## 1. Introduction

Despite the development of new treatments and therapies designed to improve the five-year survival rate, ovarian cancer still remains the deadliest cancer of the female reproductive tract [1]. Due to the 21,550 new cases and 14,600 deaths estimated by the National Cancer Institute in 2009, it also continues to be the fifth leading cause of cancer death in women throughout the United States [2]. Unfortunately, most cases are diagnosed in the late stages of the disease, when the five-year survival rates fall below 20%. Actually, less than 25% of cases are limited to the ovary alone at the time of diagnosis [3], with most patients having metastatic disease at presentation. This further contributes to worsening the prognosis. The lack of precise early warning signs is one of the factors that further contribute to the fact that only 25% of ovarian tumors are identified at stage I [4]. As most cases present in late stages of the disease, few opportunities are present for treatment and to ultimately improve survival. 

Many risk factors have been associated with the increasing prevalence of ovarian cancer; these include age (mainly perimenopausal and postmenopausal age), positive family history (5–10% of cases are familial) [5], genetics (BRCA1 and BRCA2 oncogenes), diet (mainly meats and saturated fats) [1], and other reproductive factors. Factors that have been shown to decrease the risk consist of oral contraceptive use, increasing parity, and gynecological surgeries (hysterectomies and tubal ligation) [1, 6–8]. Other elements such as breast feeding and the use of hormone replacement therapy (HRT) have demonstrated little or no effect on risk [1, 6].

## 2. Methods

A comprehensive literature review was performed in PubMed using the keywords “ovarian cancer” and “biomarkers.” The results produced were filtered by limiting the search to manuscripts which discussed studies of human subjects within the past ten years. This initial search produced 4,400 associated papers. Additional searches were further performed using the keywords cancer, cancer of female gonad, genetic markers, molecular markers, diagnostic markers, and prognostic markers to supplement the information that was obtained. 48 papers were selected for inclusion in the manuscript following careful review of the abstracts. These papers consisted of 1 meta-analysis, 10 reviews, and 37 original papers.

Subsequent searches were also performed in the gynecological book, *Ovarian Cancer: Methods and Protocols*, using the same keywords as utilized in the PubMed database. Following thorough review of the introductions (as no abstracts were available), 3 additional review papers were selected for inclusion in the manuscript.

## 3. Diagnostic Procedures in Ovarian Cancer

Despite these setbacks, early diagnosis of ovarian cancer has shown to improve the five-year survival rates to over 90%. Detection at early stage offers a potential reduction in mortality. Massive efforts have been devoted to discovering an effective screening mechanism for early-stage diagnosis prior to the onset of clinical symptoms. 

The objective of such a screening mechanism revolves around reducing mortality with an early-stage diagnosis. Currently, diagnosis of early stages of the disease is very limited, as there have been no clinically accepted tests or screening mechanisms approved for this purpose. The focus and interest of many researchers and clinicians has been drawn upon many novel diagnostic markers that may be present within early-stage ovarian tumors, as many marker panels have shown promise recently [9]. There are many challenges that prevent utilizing biomarkers as a potential screening tool. The preeminent screening methods have focused on detecting cancer before it becomes invasive through the identification of precursor lesions. This allows for the prevention of invasive ovarian cancer through early-intervention techniques. This paper will focus on current screening techniques, as well as novel biomarkers and their potential role in early detection of ovarian cancer.

### 3.1. Current Screening Techniques and Limitations in the Early Diagnosis of Ovarian Cancer and Tumor Markers as a Screening Modality

Existing screening modalities have been classified according to the first-line test. The screening approaches utilized across a large population base have centered on the use of tumor markers, ultrasound (commonly transvaginal ultrasound), or a combination of both techniques [10, 11]. Secondary tests usually follow first-line tests, on the basis of a positive or equivocal result. Applied to a smaller population base, the secondary test is typically color Doppler ultrasound if the first-line tests are tumor markers or vice versa [11]. Both tumor markers and ultrasound have various advantages and disadvantages that determine their ultimate effectiveness in screening for early-stage ovarian cancer. Utilized for multiple purposes in clinical practice such as screening asymptomatic patients, treatment planning, and disease recurrence monitoring, tumor markers have shown great promise as being mostly inexpensive, noninvasive, and with relatively fast processing times. Numerous markers can also be detected in the plasma, serum, peritoneal fluid, or in the urine. 

Despite their many practical uses, tumor markers have not obtained widespread acceptance for early detection of disease, as few markers have shown potential in overcoming the many challenges of a screening modality to enter randomized control trials [11]. Natural biological variation of certain markers may occur over time, creating a number of false-positive results. Assay measurement error may also occur [11]. Additionally, with the high costs that accompany large randomized controlled trials and the relatively low occurrence of most cancers, only the most promising markers have been evaluated further. Cancer antigen 125 (CA125) has shown to be one of those markers.

#### 3.1.1. Cancer Antigen 125

Currently, the only biomarker that has proven to detect ovarian cancer before the onset of clinical symptoms and is widely used in clinical practice is CA125 [3]. As a high-molecular-weight glycoprotein, CA125 is normally expressed in a variety of epithelial cell types. These range throughout adult tissues derived from Mullerian (endocervical, endometrial, and tubal) and coelomic (peritoneum, pericardium, and mesothelial cells of the pleura) epithelia [12]. CA125 is reported to have one of the highest sensitivity and specificity among biomarkers for ovarian cancer. Nakae et al. demonstrated that among 32 patients with ovarian cancer, 34 patients with benign ovarian tumors, and 31 healthy women, CA125 had a sensitivity of 84.4% and a specificity of 66.3% in predicting this disease [13]. Serum CA125 levels has also been proposed to reflect the relative volume of the ovarian tumor, based on the results of previous clinical trials, but this has not been reproducible across studies [11]. 

Despite the benefits accompanying the use of CA125, many challenges exist that render it not as effective in early screening. One of the primary challenges is its decline in sensitivity in early-stage ovarian cancer [14]. A number of false-positive results could also occur, since levels of this marker are naturally increased with ovulation and may be elevated with endometriosis, fibroids, and many other benign conditions; therefore, this marker is more effective in postmenopausal women. In addition, cutoff levels (30 or 35 U/mL) that are used for identifying a positive CA125 test have only been established for patients with a clinical form of the disease. This value was not recommended for screening asymptomatic patients [11].

Regardless of these challenges, CA125 has been widely used as a key screening component of the recent United Kingdom Collaborative Trial of Ovarian Cancer Screening (UKCTOS). This large randomized controlled trial is designed to evaluate the effect of screening on overall mortality. Instead of solely looking at serum levels of CA125, this trial makes use of a risk of ovarian cancer (ROC) algorithm to effectively evaluate ovarian cancer risk. Specifically, the study looked at over 200,000 postmenopausal women randomizing them to screening (100,000) versus no screening (100,000). Women within the screening group were further randomized to undergo annual transvaginal ultrasound (TVS) alone (50,000) or multimodality screening (MMS) with annual CA125 values interpreted by the ROC. In the ultrasound screening (USS) group, 845 of 48,230 women underwent surgery (1.8%) compared to 97 of 50,078 women in the MMS group (0.2%). Preliminary results have been promising, as primary invasive cancers of the ovary and fallopian tube with the MMS approach have achieved a sensitivity, specificity, and positive predictive value of 89.5%, 99.8% and 35.1%, respectively. The USS group reported sensitivity, specificity, and positive predictive value of 75%, 98.2%, and 2.8%. Interestingly, 48% of invasive malignancies (58) were diagnosed in stage I/II [15]. It will be important to determine the effect of these screening efforts on overall mortality within the coming years, and if they remain consistent in detecting the disease in its early stages.

#### 3.1.2. BRCA1/BRCA2

Screening methods that focus on identifying mutations and abnormalities in BRCA1 and BRCA2 genes have also been extensively used in current clinical practice. With mutations in these tumor suppressor genes, women have dramatically increased risks in developing ovarian cancer. By age 70, women with BRCA1 or BRCA2 mutations have been shown to have a 20–60% calculated risk of ovarian cancer [16]. However, these familial cases only account for roughly 10% of cases, as ninety percent of ovarian cancers are sporadic in nature [4]. These genes have also not been associated with certain ovarian cancer stages or histological subtypes [17]. Additionally, women less than 70 years of age only have demonstrated a 5% incidence rate in BRCA1 mutations [16]. While not as efficient as is currently believed, additional screening tests along with BRCA1/BRCA2 are needed to accurately detect the presence of ovarian tumors within patients.

#### 3.1.3. Ultrasonography

Ultrasound, specifically transvaginal sonography (TVS), is another screening technique currently in use with the potential to aide in early detection. Among 25,327 asymptomatic women who received annual TVS screenings over 18 years, one study found a sensitivity of 85%, 98.7% specificity, and a positive predictive value of 14% [18]. Supplementary studies have demonstrated similar elevated specificity and positive predictive value for ovarian cancer, noting its promising use in early screening [19, 20]. The high sensitivity noted during early stages of the disease has encouraged its use as a first-line test. 

Despite the many benefits that have been demonstrated through transvaginal ultrasound, multiple limitations prevent widespread use. The foremost limitation involves the overall cost of performing annual screenings of the entire postmenopausal population [4]. Additionally, it is felt that the majority of epithelial ovarian cancer grows rapidly and metastasizes early in disease [21]. This makes it difficult to track the progression and location of the tumor.

### 3.2. Implications of Acceptable Screening Techniques

**Figure 1:**
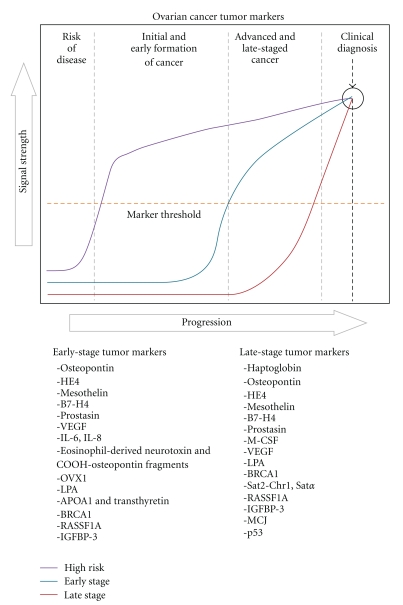
*Ovarian cancer tumor markers*. Through utilization in screening tests, signals provided prior to clinical diagnosis and symptoms help determine the value of markers in disease detection as (1) at-risk, (2) early stage, or (3) late stage and diagnostic. Markers investigated in this paper are categorized according to their marker type.

In order to overcome these challenges, it is beneficial to distinguish the various types of ovarian cancer markers. Based on a study by Urban and Drescher [22], ovarian cancer markers can be differentiated into three categories. Figure 1 depicts the behavior of these three theoretical markers throughout the development of ovarian cancer, from early risk to cancer formation, late-stage cancer, and finally clinical diagnosis. Late-stage tumor markers in Figure 1 are good diagnostic markers, as they remain elevated around the time of diagnosis. However, prior to diagnosis, these markers remain clinically undetectable in the early-stages of the disease. Useful early stage markers need to detect the disease when it is still localized. High-risk markers are elevated in high-risk patients even though there is still no disease process. Once these target patients are identified, preventive treatment, if available, can be implemented early to ensure the best possible prognosis. The associated markers that were investigated in this paper are further categorized according to their marker type (early stage or late stage) in Figure 1.

#### 3.2.1. Early-Detection Requirements

The low incidence of ovarian cancer within the postmenopausal population in Europe and the United States creates many restrictions which prevent the development of efficient and effective early screening techniques. Badgwell and Bast proposed that a useful screening mechanism must achieve a specificity larger than 99.6% to obtain a positive predictive value (PPV) greater than 10%, and a sensitivity greater than 75% [4]. The feeling among many gynecologic oncologists involved is that the numbers needed to treat (NNT) is 10 surgeries per case of ovarian cancer detected [4, 23]. Screening techniques that focus on this may be important to overcome the challenges that accompany the detection of early forms of ovarian cancer.

Previous and current studies have generally directed their focus on three methods to identify potential candidate affected genes within ovarian cancer: (1) downregulated genetic expression in ovarian cancer, (2) other cancers with epigenetic alterations in new genes and tumor suppressor genes which possess epigenetically related genes, and (3) genes with loss of heterozygosity (LOH) regions [24]. The focus of few additional studies has been on identifying hypomethylated genes. Researchers and clinicians also have been interested in various proteins, cytokines, and other factors that may be expressed throughout the disease. Prospective tumor markers within ovarian cancer can be classified further as novel serum markers and key genetic and epigenetic markers. Some of the most promising markers for the detection of ovarian cancer are included in this study. A detailed list of various studies and their findings regarding these novel tumor markers can be found in Tables 1 and 2.

## 4. Novel Tumor Markers in Ovarian Cancer

### 4.1. Haptoglobin

As an acute phase reactant protein that originates mainly from the liver, haptoglobin has been shown to be expressed in some forms of ovarian cancer within the ascitic fluid and serum as reported in several studies [62, 63]. Supplemental studies have shown its elevation in inflammation, infections, and in malignant disease such as lung cancer [64], malignant lymphoma [65], and breast cancer [66]. One study, which accounted for 66 malignant ovarian tumors, 60 benign ovarian tumors, and 10 normal healthy women, found significantly higher levels of haptoglobin in early-stage disease than among healthy controls. This may indicate its possible importance in diagnosing early forms of the disease. However, concentrations of this marker were significantly more elevated during late stages of ovarian cancer than in healthy controls or benign tumors [25].

Survival rates, outcomes, and treatment response monitoring have also been correlated with expressed levels of haptoglobin. Significantly higher levels of haptoglobin have been found in early stages of the disease and have been associated with poor prognosis [25]. Similarly, haptoglobin levels were found to decrease along with CA125 during chemotherapy [26]. However, additional studies need to be conducted to confirm the exact specificity and sensitivity of this marker and its behavior during various stages of ovarian cancer.

### 4.2. Osteopontin

Another ovarian tumor biomarker that has been associated with tumor progression and metastasis is osteopontin (OPN). Expressed as an adhesive glycoprotein and synthesized by vascular endothelial cells and osteoblasts, OPN regulates immune and inflammatory process within various cell types. This is especially true following infection or cell injury [27, 67]. Since OPN also has the ability to inhibit apoptosis, there is a positive correlation between metastatic potential and increasing OPN expression [67].

Many key studies have demonstrated the usefulness of this marker with disease monitoring following oophorectomy and in detecting recurrent ovarian cancer [13, 68]. It also may be used as a noninvasive screening test for early diagnosis, as elevated levels of OPN can be measured in the urine [43]. It was shown to be significantly elevated during advanced stages of the disease, when combined with CA125 in a biomarker screening panel, high sensitivity was achieved, reaching 93.8%, but exhibiting low specificity levels of 33.7% [13]. With a sensitivity level of 81.3% alone [13], OPN may have a lower potential than CA125 to accurately detect the presence of ovarian cancer. However, patients with significantly higher levels of OPN were noted to have a much poorer prognosis [27].

### 4.3. Human Epididymis Protein 4 (HE4)

As a component of the disulfide-core protein group, the WFDC2 (HE4) gene is also elevated in ovarian cancer [69–71]. In addition, many institutions have noted its potential as a promising marker [3]. As exhibited in Table 1, a key study noted the marker's high sensitivity and specificity in 67 ovarian cancer cases, reaching levels of 90% and 77.6% [28]. In comparison to CA125, SMRP, CA72-4, and osteopontin, HE4 possessed the highest sensitivity in detecting stage I ovarian cancer [28]. The expression of HE4 is more in endometrioid and serous ovarian cancer, possibly enabling one to distinguish among several tumor types [29]. Another reported benefit is that HE4 has less false positives, especially in nonmalignant ovarian diseases (more specific), and possessing a similar sensitivity as CA125 among blinded serum studies of women with nonmalignant disease [71, 72]. This demonstrates a possible role with CA125 in the creation of an effective biomarker panel.

HE4's role in a prospective biomarker panel for early screening has been widely studied. The most notable role of HE4 thus far is identifying cancers preoperatively using CA125 and HE4 with 94% sensitivity. The utility of screening is not well tested, and adding HE4 to CA125 for followup of previously treated ovarian cancer cases shows minimal (and not likely clinically useful) changes in sensitivity 72.9% versus 76.4% [28].

### 4.4. Mesothelin

Normally expressed on the surface of mesothelial cells lining the body cavities as the name suggests, mesothelin is a protein that has shown to be a helpful diagnostic marker of several cancers such as mesotheliomas, ovarian, and pancreatic cancers, where it is overexpressed [30]. This marker has very high specificity as well as sensitivity for patients with ovarian cancer [29].

Mesothelin has many beneficial characteristics that set it apart from other potential markers currently being studied. Its preeminent distinctiveness focuses on its temporal stability [73], which may assist in earlier diagnosis of high risk patients [71]. Additionally, when combined with CA125 as a combined marker, McIntosh et al. noted a greater sensitivity for cancer diagnosis. Mesothelin was also shown to possess comparable specificity and sensitivity to CA125 for ovarian cancer diagnosis [31]. This suggests a potential supplemental role in combination with CA125 in the monitoring and diagnosis of patients with ovarian cancer. Another benefit of this marker is that it is released into the urine, offering a new, noninvasive approach to the detection of ovarian carcinoma [71].

### 4.5. B7-H4

Shown to promote malignant transformation of epithelial cells, B7-H4 is expressed in T cells as well as ovarian carcinoma [73, 74]. This immunomodulatory protein has also been shown within serous, endometrioid, and clear cell ovarian carcinomas [34]. Appreciably, at 97% specificity, B7-H4 levels were found to be elevated within 45% of early-stage cancers [33]. Most notably, a larger portion of ovarian cancers were detected when combined with CA125 (65%) than either B7-H4 (45%) or CA125 (52%) individually.

### 4.6. Prostasin

Prostasin, generally secreted by the prostate gland as a serine protease, has been shown to be overexpressed in ovarian cancer cell lines [36]. Mok and coinvestigators discovered that this marker was strongly expressed within cancerous epithelial tissue in comparison to the normal ovary. Among 37 nonmucinous ovarian tumors and in combination with CA125, prostasin showed encouraging results in a biomarker panel. Sensitivity and specificity of 92% and 94% were noted in detection of ovarian cancer [36]. Further clarity of early-stage sensitivity still needs to be achieved.

### 4.7. Macrophage Colony Stimulating Factor and OVX1

As a cytokine that stimulates the differentiation and growth of macrophages and monocytes, macrophage colony stimulating factor (M-CSF) has been discovered to have elevated levels in ovarian cancer patients [75]. At 98% specificity, this marker was measured in 68% of ovarian cancer cases [37]. When combined with CA125, the prospective biomarker panel demonstrated sensitivity around 90% [37]. 

Promising studies have also included the use of OVX1 with M-CSF. OVX1, a modified Lewis X determinant on mucin, is significantly increased in epithelial ovarian cancer, as 70% of 93 patients had elevated levels with clinically proven disease [76]. In spite of loss in specificity, when combined with M-CSF and CA125II, sensitivity levels improved to 76% in comparison to the sensitivity of CA125II alone (66%) [44].

### 4.8. Vascular Endothelial Growth Factor (VEGF)

Shown to play an integral role in ovarian cancer pathogenesis [77], VEGF has been reported in both nonmalignant and metastatic forms of this disease [78]. Patients with stage I ovarian cancer demonstrated significantly elevated levels of VEGF when compared with benign ovarian disease. They also possessed high sensitivity values (96%), when combined with CA125, but low specificity [39]. In addition, VEGF levels were significantly elevated in advanced stages [41]. 

Other data also suggested its usefulness as a prognostic marker and for therapy monitoring. Postoperative VEGF levels were significantly lower in comparison to preoperative levels, while significantly elevated values were seen in patients with metastasis as compared to patients lacking metastasis [39]. This may be compared clinically in the near future as the latest randomized trial for advanced stage ovarian cancer in the US, incorporating anti-VEGF therapy with standard cytotoxic agents. Since specificity remained low within biomarker panels from the addition of this marker, VEGF may serve a better role as a prognostic marker in women with the disease. More research is needed to further define its role.

### 4.9. Additional Serum Markers

Validation of other biomarkers has proven difficult, even though researchers have derived many promising results. Markers such as interleukins 6 and 8, lysophosphatidic acid, eosinophil-derived neurotoxin and COOH-osteopontin fragments, apolipoprotein A1, and transthyretin have mostly noted large increases in sensitivity and specificity in several forms of ovarian cancer [41, 46, 79, 80]. Many additional studies are required to truly determine their effectiveness in early detection.

## 5. Key Genetic and Epigenetic Markers

### 5.1. Hypermethylated/LOH Genes

#### 5.1.1. BRCA1

As mentioned previously, the BRCA1 gene has been extensively studied due to its inherent role in familial ovarian and breast cancers. It is responsible for the preservation of genomic integrity and located on chromosome 17q12-21 [81]. Various studies have noted immense amounts of hypermethylation of this gene among ovarian tumors [82]. Hypermethylated BRCA1 gene has diminished expression values, ranging from 12% to 16% among epithelial ovarian cancers [17, 47]. A promising fact is that BRCA1 silencing has been discovered within early-stage disease, including some stage IA [17]. Even though common acceptance of BRCA1 mutations has been through familial linkage, LOH has been shown to occur in a vast amount of sporadic forms of the disease [81, 83]. Additionally, LOH of BRCA1 has been correlated with hypermethylation in this cancer [81]. Therefore, BRCA1 mutations may play a key role in both sporadic and familial forms. However, as this gene is hypermethylated within only a fraction of ovarian cancers, a number of additional tumor suppressor genes and other genes are needed to effectively detect all forms of the disease.

Recent studies with BRCA1, however, have noted a promising role in determining clinical outcome for ovarian cancer patients. Hypermethylation of BRCA1 strongly associates with the loss of BRCA1 protein and RNA [84]. This is significantly associated with a poor patient outcome [47]. These studies help demonstrate that BRCA1 hypermethylation may prove as a minimally invasive approach for predicting patient response to standard therapies, especially since it has been found within ovarian cancer patient serum [53].

#### 5.1.2. Ras Homologue Member 1 (ARHI)

Located on chromosome 1p31, this tumor suppressor gene has shown LOH in roughly 40% of ovarian carcinoma [48, 85]. Normally, ARHI is expressed in consistent amounts within normal ovarian epithelial cells. This expression is mainly lost upon the formation of ovarian cancer [48]. Interestingly, the parental allele that remains is silenced in 10–15% of patients with the disease [86, 87]. ARHI has also been significantly associated with serous and endometrioid forms as well as a prolonged disease-free outcome, despite having no correlation with any stage or grade of the tumor [88]. However, supplementary studies are required to truly confirm this characteristic.

#### 5.1.3. OPCML

Demonstrated to be hypermethylated or have LOH in 27–49% of ovarian cancer cases, opioid binding protein/cell adhesion molecule-like gene (OPCML) is shown to reside at chromosome 11q25 [49]. One study found a significant amount of methylated OPCML in early stages of the disease. However, tumor grade, stage, and histological type had no influence on the presence of methylation [89]. Due to small sample sizes and lack of many large studies in this area, more research is needed to correlate expression level of this gene with ovarian cancer.

#### 5.1.4. RASSF1A

Not only has RAS association domain family 1A gene (*RASSF1A*), a tumor suppressor gene, been identified in ovarian cancer, but also in lung, renal cell, colon, and breast cancers [90]. Located at chromosome 3p21, Yoon et al. found methylation rates of this gene as high as 40%. This characteristic is significant, as normal tissue samples did not show gene methylation [90]. Its usefulness in a biomarker panel was demonstrated when combined with BRCA1. Hypermethylation improved to 68% and was seen across all histological types, grades, and stages that were examined [53]. Due to its usefulness in a small genetic marker panel, further studies should be looked at to determine its true effectiveness with other markers (i.e., CA125) in detecting ovarian carcinoma.

#### 5.1.5. Insulin-Like Growth Factor Binding Protein 3 (IGFBP-3)

As one of the most abundant IGF binding proteins noted in the serum, IGFBP-3 is typically shown to regulate the apoptosis and mitogenesis suppression properties of IGF proteins [54, 91]. Studies have shown detection of IGFBP-3 promoter methylation in 44% of epithelial ovarian cancer cases [54]. Patients with this form of ovarian cancer have also displayed lower serum levels of this protein in comparison to healthy individuals [92]. Significantly higher levels of methylation were noted in early-stage disease and associated with overall survival outcome [54]. It remains unknown if this marker could be used as a prognostic marker in late-stage patients.

#### 5.1.6. 14-3-3sigma (SFN)

A key characteristic that has distinguished SFN is its ability to characterize the histological forms among various types of epithelial ovarian cancer. Among many ovarian cancer cell lines that were composed of different histological forms (clear cell, serous, endometrioid, and mucinous), Kaneuchi et al. discovered higher frequencies of methylation (78.6%) among clear cell ovarian tumors when compared to other histologic subtypes of epithelial tumors. Lower expressions of this protein were demonstrated, suggesting 14-3-3sigma gene inactivation through methylation. Protein expression was dramatically elevated in other histological types (89.5% serous, 90% endometrioid, and 81.8% mucinous) as methylation rates widely differed among each form [55]. One limitation could be 14-3-3sigma expression being elevated among both normal controls and 73.5% of ovarian cancer cases [93]. While certain treatment regimens may be more effective with the knowledge of the histological type of ovarian tumor, the presence of ovarian cancer will need to be confirmed through the use of other markers or screening techniques prior to this marker's use.

### 5.2. Hypomethylated Genes

#### 5.2.1. Metastasis-Related Gene Synuclein Gamma (SNCG)

Found on chromosome 10 and not expressed in normal ovarian epithelial tissue, SNCG has been generally expressed in advanced and aggressive ovarian cancer [50, 86]. Also expressed in breast cancer and thought to have a similar role in ovarian cancer, SNCG causes significant cellular proliferation and differentiation [50]. Studies, however, have varied in the degree of hypomethylation found, ranging from 75.7% to 100% [50, 51]. To our knowledge, no additional correlation has been made relating to the clinical stage or histological tumor type and promoter SNCG hypomethylation. More extreme studies are needed to prove the exact worth of this marker.

#### 5.2.2. Satellite 2 DNA (Sat2): Chromosome 1 (Chr1), Satellite Alpha (Sat*α*)

More prevalent in high grade and advanced stage tumors, Sat2 DNA has shown high degrees of hypomethylation among adjacent heterochromatin regions of chromosome 1 in ovarian cancer [94]. Hypomethylation levels reached 30% (Chr1, Sat2) and 33% (Chr1 Sat*α*) amid 115 ovarian cancer specimens [52]. Serous and endometrioid ovarian tumor forms could be distinguished with elevated hypomethylation levels than other histological types [52]. Interestingly, a connection between tumor stage and different Sat2 DNA hypermethylation has been reported [94].

#### 5.2.3. DNAJ (MCJ)

The MCJ gene, DNA methylated and silenced within normal cells, may be regarded as a useful marker in chemotherapy treatment response monitoring among ovarian cancer patients. This gene (mapped to chromosome 13q14.1), when expressed in ovarian cancers, has been identified to render epithelial cells to become more sensitive in response to many mainstream chemotherapeutic agents (i.e., paclitaxel and cisplatin) [56, 95]. Among high levels of methylation (>90%) in 17% of ovarian cancer cases, Strathdee and his colleagues associated a poorer survival rate and response to chemotherapy [56]. These high levels of methylation have been linked to the lack of expression of the MCJ gene [95]. While the prognostic value of this marker remains high, more studies are required to characterize the chemosensitivity correlation.

#### 5.2.4. p53

Involved in cell cycle regulation, p53 is regarded as one of the most widely studied tumor suppressor genes. Mutations in this gene have been shown in 50% of ovarian carcinomas [16]. Benefits of the p53 marker use include measuring metastatic potential [96] and distinguishing high-grade serous histological ovarian tumors from other ovarian cancer types [97]. Since allele loss and mutations have been demonstrated across all stages of ovarian tumors [97], it may prove effective in earlier detection of this disease. As only 50% of mutations within this gene occur in ovarian cancer patients, this may still continue to be the key barrier in widespread use of this marker for ovarian cancer detection.

#### 5.2.5. ARID1a

ARID1a, a key epigenetic regulatory gene, recently has drawn large interest due to its demonstrated involvement in both clear cell and endometrioid ovarian cancers. Published in September 2010, two crucial studies showed that mutations in the ARID1a were found in roughly half of clear cell ovarian cancers tested. Wiegand et al. stated that ARID1a mutations were seen in 55 of 119 ovarian clear cell carcinomas (46%), in addition to 10 of 33 endometrioid carcinomas (30%) [58]. ARID1a mutations were also found in 24 of 42 clear cell ovarian carcinomas tested (57%) [59]. From these discovered mutations, both studies have suggested ARID1a serving a role as a tumor suppressor gene with a focus on remodeling chromatin via unwinding DNA [58, 59]. When this gene is unable to function properly, it may lead to cancer formation.

Despite these fascinating results, it will be interesting to view if these two forms of ovarian cancer continue to show mutations in this gene. More studies should be done to identify this defect in these ovarian cancer forms, determining its use as an effective detection marker. Studies should also focus on the epigenomes of these two cancer forms, noting any differences that may occur despite mutations in the ARID1a gene. As suggested by one researcher, by having a better understanding of the epigenomes of these two ovarian cancer forms, it may lead to better epigenetic therapy in the future [98].

## 6. Proteomics and Metabolomics

Several of the potential ovarian cancer tumor markers have been identified due to the new technologies and techniques that have been derived through the fields of proteomics and metabonics. Their importance lies in the pathophysiology of cancer, as the genetic defect is transcribed and translated into proteins, ultimately leading to the abnormal phenotype [99]. These fields have ushered in new promises, ranging from the discovery of ovarian cancer in its early stages to treatment modality monitoring and guidance. 

Techniques in these fields have focused on fluid and tissue screening. In these screenings, focus is drawn on identifying the presence of disease, or characterizing known malignant cancer samples [100]. The traditional approach utilizes mass spectroscopy (MS) to evaluate serum samples for diagnostic markers. New technological approaches have expanded techniques to include liquid chromatography, matrix-assisted laser desorption and ionization, and surface-enhanced laser desorption and ionization to screen serum, urine, and ascites for potential markers of early ovarian cancer [100]. Through these screenings, detection of disease recurrence may also have a correlation with the presence of certain markers.

Unfortunately, several issues have been raised utilizing these methods to help determine potential biomarkers. Studies have shown that protein concentrations where potential markers may exist for early detection are 10^6^-10^7^ less than that of plasma proteins [101]. Further attempts that are designed to decrease plasma concentrations may also decrease potential markers that are bound to albumin [102]. However, several researchers have reported high sensitivities and specificities for potential markers using traditional MS practices between healthy and cancer-ridden serum samples [103, 104]. It will be interesting to see how these techniques translate to predicting the presence of ovarian cancer in patients.

As discussed previously, several potential tumor markers have been investigated in several ovarian cancer forms. Most of these markers that were confirmed and validated in further studies were classified into plasma proteins (apolipoprotein) and acute phase reactant proteins (transthyretin, haptoglobin, etc.) [105]. Unfortunately, these markers have been present in a variety of diseases and conditions, decreasing their specificity and desirability as potential biomarkers for ovarian cancer.

Clinically, trials are currently underway to identify proteomic patterns that can diagnose patients with ovarian carcinoma. A Gynecologic Oncology Group Trial (GOG-220) of over 2,000 women has this goal. This study will look at serum samples of women with an undiagnosed pelvic mass who are undergoing surgical intervention [100]. Goals of this study include identifying proteomic patterns that can differentiate between benign or nonovarian malignancies and malignant ovarian cancer in presurgical serum collections. Other goals consist of utilizing these patterns to distinguish between early- and late-stage disease and determining disease prognosis [100]. Results of this trial have not yet been reported. Larger clinical trials with larger sample sizes are truly needed both for the discovery and validation of potential markers and patterns for ovarian cancer detection. This may help reduce false positive rates and help hone in on predicative diagnostic markers that can apply to many forms of the disease.

## 7. Biomarker Panels in Detection of Ovarian Cancer

Many promising predictive tumor markers have been evaluated in patients with ovarian cancer. These markers have been evaluated in combination with one another to improve the sensitivity, specificity, and positive predictive value of the test, as 20% of ovarian cancers have been noted to express little or no amounts of CA125 [106]. One key panel focuses on combinations of nine markers, including CA125, SMRP, HE4, CA72-4, activin, inhibin, osteoponin, EGFR, and ERBB2 among 233 women who had ovarian cancer. Results from this study showed that the combined markers of CA125 and HE4 had a greater sensitivity than either marker alone [28]. Another panel of six tumor markers also improved the sensitivity and specificity of CA125 alone (72%, 95%) to 95.3% and 98.7%. In one study, 221 of 224 women in a test group that included 43 women with ovarian cancer were classified appropriately (98.7%) [107].

Data from another study with a four-marker panel (CA125, apolipoprotein A-1, transthyretin, and transferrin) reported a sensitivity and specificity of 96% and 98%, respectively, for early-stage ovarian cancer [108]. Additionally, another panel test showed ovarian cancer sensitivity and specificity of 91.3% and 88.5% [109]. These panels, however, have yet to be validated in clinical trials.

While specificity has shown to lack in terms of single gene methylation assessment, a common belief is that sufficient accuracy may be achieved for population screening through the use of multiple methylation markers [110]. Several studies have supported this belief. Detection of ovarian cancer in high-risk patients was significantly predicted through the use of a methylated five-gene panel [111]. Progression-free survival of ovarian cancer was associated with one panel consisting of over 100 methylated DNA markers [112]. While these markers may assist CA125 in improving the overall specificity of ovarian cancer detection, more studies are needed to confirm this fact. Further investigation is needed to evaluate the total worth of marker panels as a screening and detection modality before implantation into clinical practice.

## 8. FDA-Approved Screening Tests (CA125, HE4, and OVA1)

Some of the key screening tools that are utilized in current practice involve identifying elevations of markers such as CA125 and HE4, as discussed previously. These tools are frequently used either in combination with each other, or even with the application of transvaginal ultrasound for detection of ovarian disease.

One of the newest serum-based tests (approved in 2009) is the OVA1 test. The key purpose of this test was to identify ovarian cancer risk in women who presented with an adnexal mass and were planning surgery. Five significant proteins were measured in the serum (CA 125-II, transthyretin, apolipoprotein A1, beta 2 microglobulin, and transferrin) and combined with an algorithm to yield an overall OVA1 score [113]. This score differed depending on menopausal status, differentiating patients either into a low- or high-risk group. Among 516 eligible women in the initial trial, OVA1 was found to aide in the presurgical assessment of patients. Sensitivity improved from 72.2% to 91.7% for nongynecologic oncologists and from 77.5% to 98.9% for gynecologic oncologists. Negative predictive values were 93.2% for nongynecologic oncologists, and 97.6% for gynecologic oncologists [114]. Overall, OVA1 was shown to have detected the majority of ovarian cancers that were missed by preoperative assessment alone. Currently, publication of this trial is still pending.

OVA1 has not yet been tested in screening patients for early-stage ovarian cancer. On the surface, it looks very similar to other biomarker panels that have been already studied. Even though this panel focused on women who presented with adnexal masses, a correlation in the panel's applicability should not yet be drawn to women who present with no masses until studied further. As suggested by one physician, consumer pressure will most likely occur to use it as a screening tool. This may lead to more expensive interventions and greater amounts of false-positive results [114]. 

The OVA1 panel also has shown some limitations, discouraging its use as a potential screening modality. Assay interference has occurred in triglyceride levels greater than 4.5 g/L and rheumatoid factor levels of at least 250 international units/mL. The cost alone has been high, valued at roughly $650 per patient [114]. Larger studies showing high predictive values are needed to justify this cost prior to its use in clinical practice as a screening tool.

Currently, many centers offer ovarian cancer screening through CA125 measurements and annual transvaginal ultrasound. Unfortunately, many efficacy studies have been conflicting, and recent studies have demonstrated ovarian cancer screening less effective, even in high-risk populations [115, 116]. One large cohort study even noted when combining both screening modalities among 341 asymptomatic women, that sensitivity and specificity were only 66.7% and 82.9%, respectively. No cancers were detected at first screening episodes. In addition, among four women who had ovarian cancer, each had a normal screening episode prior to diagnosis [117]. Based on this and other studies, only utilizing these two modalities for ovarian cancer screening may prove ineffective with a high rate of false-negative results and poor sensitivity.

As with all FDA-approved screening tests, more clinical studies are needed to determine their true predictive values in the early detection of ovarian carcinoma. While each one of these approved tests have shown some benefit, they should never be used by themselves in place of clinical judgment. Clinical evaluations including risk assessment should be combined with additional screening tests (i.e., biomarker panels and transvaginal ultrasound) to ensure the greatest sensitivity and specificity possible. Supplemental research should also center on combinations of these tests and their predictive values, helping providers better understand the most effective method for ovarian cancer detection.

## 9. Future Implications, Direction, and Improvements

New and innovative approaches will still be needed to detect preclinical disease. Biomarkers continue to have great potential in serving as an efficient screening tool in the early detection of ovarian cancer. However, mismanagement, overuse, and rising costs of certain screening techniques have created many problems in effectively detecting early forms of this disease. These tests have often lacked cost effectiveness, as many women who develop ovarian cancer lack significant risk factors. This leads to a need for screening of all asymptomatic women for the testing to be effective. In order to perfect and establish an efficient widespread screening modality within the general public, further refinements need to be made among the screening tests through randomized controlled trials. More resources and tests should also be sought, while screening all women who may be at high risk for developing ovarian cancer.

Some authors have suggested implementing risk calculations to determine the efficient allocation of screening resources [11]. These calculations are based on the baseline CA125, the patient's age upon tumor development, CA125 elevation rate following tumor development, and expected variation in CA125 levels. Ideally, CA125 would be measured annually, since ovarian cancer commonly presents as a rapidly growing tumor. By observing trends over time of CA125, women with stable levels can be ruled out, increasing the overall specificity. When putting these principles into practice in 22,000 postmenopausal UK women over the age of 45, Skates et al. demonstrated a high level of specificity, while increasing early detection sensitivity to 86% from 70% [11]. Newer data among 202,638 postmenopausal UK women between ages 50 and 74 showed similar high levels of specificity and sensitivity: 98.2–99.8% and 84.9–89.4%, respectively [15]. While refinement and testing of this approach is still needed within large-scale trials, a combination of many biomarkers and early screening modalities may be the key to obtain the most accurate forms of ovarian cancer detection.

In terms of clinical setting, practical screening techniques need to focus on biomarkers that demonstrate temporal stability and are relatively noninvasive to obtain. Approaches and tests that look at markers found in the urine (mesothelin) or serum may help identify more clinically relevant markers. This would help reduce the need to obtain tissue samples from normal and ovarian cancer patients for detection of this disease. Similarly, markers that show long-term stability may aid in determining risk and ultimately improve the overall sensitivity and specificity of ovarian cancer detection.

Identifying early forms of ovarian carcinoma and a patient's prognosis may have a greater chance of being detected through the combination of many biomarkers that should be studied further. Among the promising markers investigated, HE4 and mesothelin are some of the best markers that have enhanced the use of CA125 due to the high sensitivity and specificity of these combination panels [28]. Additionally, panels which included the MCJ gene and VEGF, both shown to be related to prognosis, may accurately detect patient survival outcome. Ultimately, the goal will be to determine the behavior of complementary markers with key markers to help improve the sensitivity as well as maintain the specificity of detecting early-stage ovarian cancer and determining disease prognosis.

## 10. Conclusions and Emerging Trends in Biomarkers for Ovarian Cancer

The ultimate aim of effective screening techniques is to bring about a reduction in mortality from ovarian cancer. As early detection continues to be vital in ovarian cancer patients, biomarkers may hold the key to unlocking effective screening strategies for the general population. It is also important to identify screening techniques with low false positive rates and high positive predictive value, so that the number of negative surgical interventions can be minimized. Since our currently available single markers are not highly sensitive or specific, a combination of markers may be utilized as a profile for risk assessment. The current problem with screening panels is that the improvement in sensitivity usually correlates with a decrease in specificity, making the target positive predictive value hard to obtain. The multimodal screening profiles of the genetic markers could be utilized in the future for risk assessment, early diagnosis, prognosis, and response to therapeutic treatment. Recent literature reports state that the screening is only recommended for the high-risk population identified as those with a family history of the disease, women with BRCA1 and BRCA2 mutations, or with hereditary nonpolyposis colorectal cancer. Recent literature reports also emphasize that the different subtypes of ovarian cancer may have different genetic biomarker expression profiles. Current randomized controlled screening trials are directed towards finding the best molecular and genetic markers for the specific histology of the ovarian tumor with the most impact on reduction in morbidity and mortality. The tumor markers identified in these trials may also lead to novel targets for antitumor therapy.

## Figures and Tables

**Table 1:** Characteristics of novel ovarian cancer serum markers across various key studies.

Marker	Sample size	Sensitivity/Specificity	Stage of ovarian cancer	Types of ovarian cancer	Additional comments	Reference
*Haptoglobin*	(1) 66M/60B/10H (2) 24M/11H	(1) Not provided (2) Not provided	(1) Late stage (2) Late stage (FIGO* stage III)	(1) Serous, mucinous, endometrioid (2) Not provided	(1) CRP levels significantly correlated with haptoglobin levels (2) Haptoglobin levels decreased following chemotherapy	(1) [25] (2) [26]

*Osteopontin*	(1) 40M (FIGO stage III) (2) 32M/34B/30G/31H (3) 67M/166B	(1) Not provided (2) 81.3%/54.7%, 93.8%/87.4%^#^ (3) 90%/19.6%, 95%/7.6%, 98%/4.9%, 90%/14.8%^*∧*^, 95%/14.7%^*∧*^, 98%/7.6%^*∧*^	(1) Late stage (2) Late stage (3) Early and late stage	(1) Not provided (2) Serous, endometrioid (3) Serous	(1) Poor prognosis^$^	(1) [27] (2) [13] (3) [28]

*HE4*	(1) 67M/166B (2) 37M/19B/65H	(1) 90%/77.6%, 95%/72.9%, 98%/64.2%, 90%/80.7%^#^, 95%/76.4%^#^, 98%/71.6%^#^, 90%/46.2%^*∧*^, 95%/45.9%^*∧*^, 98%/30.8%^*∧*^ (2) 80%/96%	(1) Early and late stage (2) Not provided	(1) Serous (2) Serous, endometrioid	(1) Highest sensitivity in detecting stage I ovarian cancer^&^	(1) [28] (2) [29]

*Mesothelin (SMRP)*	(1) 21M/24H (2) 52M/43B/220H (3) 30M/68H	(1) Not provided (2) 60%/98% (3) 77%/100%	(1) Late Stage (2) Early stage and late stage (3) Late stage	(1) Not provided (2) Not provided (3) Not provided	(2) Greater fraction of ovarian cancer detected with CA125 than either marker alone	(1) [30] (2) [31] (3) [32]

*B7-H4*	(1) 236M/150B/ 260H (2) 326M/43B/32H	(1) 65%^#^/97%^#^ (2) Not provided	(1) Early stage (2) Late stage	(1) Serous, endometrioid (2) Serous, endometrioid, clear cell	(2) 75% of tumors were positive for one or both markers (w/CA125)	(1) [33] (2) [34]

*Prostasin*	(1) 12M (2) 64M/137H	(1) Not provided (2) 51.4%/94%, 92%^#^/94%^#^	(1) Late stage (2) Early and late stage	(1) Not provided (2) Nonmucinous	(2) Sensitivities and specificities based on 37 nonmucinous ovarian cancers	(1) [35] (2) [36]

*Macrophage colony stimulating factor (M-CSF)*	(1) 69M/80H (2) 69M/55B/634H	(1) 68%/98%, 90%^#^ (Sensitivity) (2) Not provided	(1) Not provided (2) Late stage	(1) Not provided (2) No difference noted		(1) [37] (2) [38]

*Vascular * *endothelial growth factor (VEGF)*	(1) 120M/25B/90H (2) 101M/34B	(1) 77.1%/87% (2) 74%/71%, 96%^#^/39%^#^	(1) Late stage (2) Early and late stage	(1) No difference noted (2) Not provided	(1) Additional data suggest usefulness as a prognostic marker and for therapy monitoring (2) Shown to be significantly associated with survival	(1) [39] (2) [40]

*Interleukins (IL-6, IL-8)*	(1) 94M/37B/80H (2) 44M/37P/45H	(1) 87.5%^+^/98%^+^ (2) 84%^=^/95%^=^	(1) Early stage (2) Early stage	(1) Not provided (2) Not provided		(1) [41] (2) [42]
*Eosinophil-derived neurotoxin and COOH-osteopontin fragments*	(1) 128M/52B/188H	(1) 72%/93%	(1) Early stage	(1) Serous, mucinous, endometroid, clear cell		(1) [43]

*OVX1*	(1) 204M/77B/117H	(1) 76%^“^/Decline in specificity	(1) Early stage	(1) Endometrioid, mucinous	(1) OVX1 alone does not increase the sensitivity to the combination of CA-125 and M-CSF for identifying patients with ovarian carcinoma	(1) [44]

*Lysophosphatidic acid (LPA)*	(1) 117M/27H	(1) 91%/96%	(1) Early and late stage	(1) Not provided	(1) Statistically significant differences between preoperative and healthy control levels, pre- and postoperative levels	(1) [45]

*Apolipoprotein A1 (APOA1) and transthyretin*	(1) 42M/65B/76H	(1) 52.4%/96.5%	(1) Early stage	(1) Serous		(1) [46]

M: cases of ovarian cancer, B: benign ovarian tumor, G: other gynecological cancers, P: benign pelvic tumors, H: healthy individuals.

*Federation of Obstetrics and Gynecology.

^$^32 patients with significant increases in osteopontin.

^#^When combined with CA125.

^*∧*^Benign disease versus stage I ovarian cancer assay sensitivity.

^&^In comparison to CA125, SMRP, CA72-4, and osteopontin.

^+^IL-8, anti-IL-8 antibodies, and CA125.

^=^IL-6, IL-8, epidermal growth factor (EGF), VEGF, monocyte chemoattractant protein-1 (MCP-1), and CA125.

^“^When combined with CA125II and M-CSF.

**Table 2:** Characteristics of major ovarian cancer genetic and epigenetic markers across various key studies.

Epigenetic marker	Location	Sample size	Cause of over and underexpression	Type of change	Percentage (%) of cases found	Stage of ovarian cancer	Type of ovarian cancer	Comments	Reference
*BRCA1*	17q21	(1) 98M/12H (2) 50M	(1) Epigenetic (2) Epigenetic	(1) Hypermethylation (2) Hypermethylation	(1) 12% (2) 16%	(2) Early and late stage	(1) Serous (2) No difference noted		(1) [47] (2) [17]
*ARHI*	1p31	(1) 38M/3C	(1) Epigenetic	(1) LOH	(1) 41%	(1) Not provided	(1) Not provided		(1) [48]
*OPCML*	11q25	(1) 118M	(1) Epigenetic	(1) Hypermethylation/ LOH	(1) 27–49%	(1) No difference noted	(1) Not provided		(1) [49]
*Metastasis-related gene synuclein-gamma (SNCG)*	10q23	(1) 5C (2) 43M	(1) Epigenetic (2) Epigenetic	(1) Hypomethylation (2) Hypomethylation	(1) 100% (2) 76.7%	(1) Not provided (2) No differences noted	(1) Not provided (2) No differences noted	(1) Reexpressed genetic expression in aggressive ovarian cancer lines (2) Expressed in a large portion of malignant tumors	(1) [50] (2) [51]
*Satellite 2 DNA (Sat2)—chromosome 1 (Chr1), satellite alpha (Sat*α*)*	Near centromeres of chromosomes 1 and 16	(1) 115M/26B	(1) Epigenetic	(1) Hypomethylation	(1) 30% (Chr1 Sat2), 33% (Chr1 Sat*α*)	(1) Late stage	(1) Serous, endometrioid	(1) More pravelent in high-grade tumors	(1) [52]
*RASSF1A*	3p21.3	(1) 50M	(1) Epigenetic, genetic^*∧*^	(1) Hypermethylation	(1) 50%, 68%*	(1) Early and late stage	(1) Serous, endometrioid, clear cell	Genetic cause: deletion^*∧*^	(1) [53]
*Insulin-like growth factor binding protein 3 (IGFBP-3)*	7p13-p12	(1) 235M	(1) Epigenetic	(1) Hyper/promoter methylation	(1) 44%	(1) Early and late stage	(1) No difference noted	(1) Significant higher levels of methylation within early-stage disease and association with overall survival	(1) [54]
*14-3-3sigma (SFN)*	1p36.11	(1) 54M/3C	(1) Epigenetic	(1) Hypermethylation	(1) 78.6% (clear cell), 36.4% (mucinous), 20% (endometrioid), 26.3% (serous)	(1) Not provided	(1) Mostly clear cell	(1) Significantly related to the pathologic type of ovarian cancer	(1) [55]
*DNAJ (MCJ)*	13q14.1	(1) 41M (stage III/IV)	(1) Epigenetic	(1) Hypomethylation	(1) 93%—some level of methylation, 17%—high level of methylation	(1) Late stage	(1) Not provided	(1) High levels of CpG island methylation correlated significantly with poor response of patients' tumors to therapy and poor overall survival	(1) [56]
*P53*	17q13	(1) 70M	(1) Genetic	(1) Allelic loss and mutations	(1) 31–39%	(1) Not provided Usually seen in advanced stage^#^	(1) Mostly serous		(1) [57]
*ARID1a*	1p35.1	(1) 119M (clear cell)/33M (endometrioid) (2) 42M	(1) Epigenetic (2) Epigenetic	(1) Allele mutation (2) Allele mutation	(1) 46% (clear cell), 30% (endometrioid) (2) 57%	(1) Not provided (2) Not provided	(1) Clear cell, endometrioid (2) Clear cell	(1) Correlated with loss of BAF250a protein (2) 7% of cases showed mutations in PPP2R1A gene	(1) [58] (2) [59]

M: cases of ovarian cancer, B: benign ovarian cancer, C: ovarian cancer cell lines, H: healthy individuals, LOH: loss of heterozygosity.

*When combined with BRCA1.

^*∧*^[60].

^#^[61].
